# Decellularised extracellular matrix decorated PCL PolyHIPE scaffolds for enhanced cellular activity, integration and angiogenesis†

**DOI:** 10.1039/d1bm01262b

**Published:** 2021-09-23

**Authors:** Serkan Dikici, Betül Aldemir Dikici, Sheila MacNeil, Frederik Claeyssens

**Affiliations:** Department of Bioengineering, Izmir Institute of Technology Izmir 35430 Turkey serkandikici@iyte.edu.tr; Department of Materials Science and Engineering, University of Sheffield, Kroto Research Institute Sheffield S3 7HQ UK f.claeyssens@sheffield.ac.uk

## Abstract

Wound healing involves a complex series of events where cell–cell and cell-extracellular matrix (ECM) interactions play a key role. Wounding can be simple, such as the loss of the epithelial integrity, or deeper and more complex, reaching to subcutaneous tissues, including blood vessels, muscles and nerves. Rapid neovascularisation of the wounded area is crucial for wound healing as it has a key role in supplying oxygen and nutrients during the highly demanding proliferative phase and transmigration of inflammatory cells to the wound area. One approach to circumvent delayed neovascularisation is the exogenous use of pro-angiogenic factors, which is expensive, highly dose-dependent, and the delivery of them requires a very well-controlled system to avoid leaky, highly permeable and haemorrhagic blood vessel formation. In this study, we decorated polycaprolactone (PCL)-based polymerised high internal phase emulsion (PolyHIPE) scaffolds with fibroblast-derived ECM to assess fibroblast, endothelial cell and keratinocyte activity *in vitro* and angiogenesis in *ex ovo* chick chorioallantoic membrane (CAM) assays. Our results showed that the inclusion of ECM in the scaffolds increased the metabolic activity of three types of cells that play a key role in wound healing and stimulated angiogenesis in *ex ovo* CAM assays over 7 days. Herein, we demonstrated that fibroblast-ECM functionalised PCL PolyHIPE scaffolds appear to have great potential to be used as an active wound dressing to promote angiogenesis and wound healing.

## Abstract 2

Decellularisation of *in vitro* generated extracellular matrix (ECM) provides an effective way to stimulate angiogenesis and wound healing.

## Graphical abstract

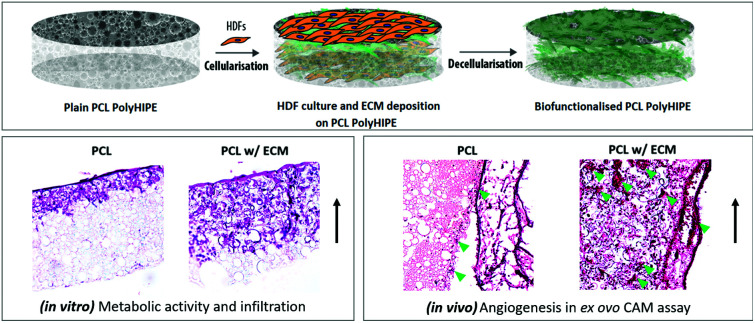

## Introduction

Skin serves as a physical barrier between the body and the external environment. It prevents microorganism invasion,^1^ mechanical, chemical, and thermal damage, and damage from exposure to ultraviolet (UV) or dehydration.^2,3^ Skin is vulnerable to traumas such as burns, scalding, and sharp cuts,^4^ and wounding can be either as simple as the loss of the epithelial integrity or deeper and more complex, reaching to subcutaneous tissues, including blood vessels, muscles and nerves.^5^ Wound healing is a complex series of events involving haemostasis, inflammation, granular tissue formation, proliferation, neovascularisation and remodelling.^6^ When the skin is wounded, the wound site needs to be covered immediately using appropriate wound dressings depending on the type of wound.^7,8^

An ideal wound dressing should provide a safe barrier layer to ensure wound cover, preserve a moist environment, be capable of absorbing any exudate from the wound surface, and create an environment that is encouraging to wound healing.^9,10^ Conventional wound dressings, such as cotton and gauze, are cost-effective and highly absorbent, but they are passive dressings and do not promote the healing process.^11^

The neovascularisation of the wound healing area is crucial and involves complex angiogenic phases of clotting and granulation before capillaries begin invading the healing site. During the haemostatic and proliferative phase of wound healing, a large number of pro-angiogenic factors, including vascular endothelial growth factor (VEGF), transforming growth factor-beta (TGF-β), platelet-derived growth factor (PDGF) and basic fibroblast growth factor (bFGF), are released to promote angiogenesis.^12–14^ The ECs are responsive to these pro-angiogenic factors as well as to extracellular matrix (ECM) molecules. Capillaries from the surrounding tissues start to invade the formed clot and within a couple of days a microvascular network forms within the area of healing.^15^ Angiogenesis is essential for wound healing as it has a key role in supplying oxygen and nutrients during the highly demanding proliferative phase and transmigration of inflammatory cells to the wound area.^16^

The use of a variety of polymers, including polylactic acid,^17,18^ polyhydroxyalkanoates,^19,20^ polycaprolactone (PCL),^21–23^ and polyvinyl alcohol^24,25^ as wound dressings materials, has been reported previously. Among these, PCL is a biocompatible and bioresorbable synthetic polymer that is FDA approved for several biomedical applications such as medical sutures and drug delivery. In addition, PCL has been reported to be used in tissue engineering (TE) as scaffolds for both hard and soft TE due to its ease of fabrication in different forms.^26^ However, one of the main challenges of PCL, and other synthetic polymers, is its hydrophobicity, which limits the cell–material interactions.^27,28^ We have previously shown the efficiency of several techniques to improve cell–material interaction including plasma treatment,^29,30^ gelatin coating,^31,32^ decoration with *in vitro* generated ECM,^33^ and co-culture with other cell types.^31^ Among these techniques, decellularisation of an *in vitro* generated ECM has considerable potential as it naturally includes more complex ECM molecules and bioactive cues.^34^ In recent decades, decellularised *in vitro* generated ECMs have been reported to be used for several biomaterial applications, such as for bone TE,^33,35,36^ cardiovascular TE,^37^ as *in vitro* models,^38^ and as bio-inks for 3D bioprinting applications.^39^ Although it has been widely used in the field of TE, the potential use of cell-derived ECM in wound healing and angiogenesis studies is very limited.

Accordingly, in this study, we aimed to develop a bio-functionalised PCL polymerised high internal phase emulsion (PolyHIPE) scaffold by combining emulsion templating and decellularisation techniques to be used as a bioactive wound dressing. First, photocurable PCL was synthesised in house, and the PCL PolyHIPE scaffolds were fabricated *via* the technique of emulsion templating, which has previously been extensively reviewed.^40^ The constructs were initially cellularised with human dermal fibroblasts (HDFs) to decorate them with an *in vitro* ECM. *In vivo*, fibroblasts and fibroblast-derived ECM plays an important role in wound healing^41^ and angiogenesis.^31^ The scaffolds were decellularised to obtain bio-functionalised scaffolds made of PCL and fibroblast-derived ECM. These bio-functionalised PCL PolyHIPE constructs were then evaluated for their performance in improving the metabolic activity of three different types of cells, fibroblasts, keratinocytes, and endothelial cells, which all actively take part in the wound healing process. Following the *in vitro* evaluation of the biological performance, the angiogenic potential of the scaffolds was further assessed using an *ex ovo* CAM assay.

This study makes a significant contribution to the world of wound management materials. Its advantages over many other biomaterial development techniques can be summarised as follows: (i) ECM decoration of PCL PolyHIPE is a promising way to improve the biological performance of synthetic patches, not only to increase the biocompatibility but also to enhance collagen deposition and to enable the induction of angiogenesis *in vivo*. (ii) The biohybrid design provides an opportunity to combine the advantages of a synthetic material (PCL), such as tunability of mechanical and physical properties and ease of processability, with the biological complexity of ECM. (iii) The use of fibroblasts to produce their unique ECM is an effective backdoor route to include the complex biochemical cues that take key roles in angiogenesis and wound healing without the addition of individual biological components. (iv) This proof-of-concept study demonstrates an effective way to develop pro-angiogenic biomaterials for the management of wounds that do not heal in a timely manner, and (v) the use of patient's own cells to provide bioactivity to PCL PolyHIPE does not possess the risk of immune rejection upon implantation.

## Materials

Pentaerythritol (98%), ε-caprolactone, tin(ii) 2-ethylhexanoate, triethylamine (TEA), methacrylic anhydride (MAA), photoinitiator (PI) (2,4,6-trimethylbenzoyl phosphine oxide/2-hydroxy-2-methylpropiophenone blend), fungizone, fetal calf serum (FCS), penicillin/streptomycin (PS), l-glutamine, trypsin, 37% formaldehyde (FA) solution, resazurin sodium salt, glutaraldehyde, ethanol, hexamethyldisilazane (HMDS), perchloric acid, picric acid, hematoxylin solution, eosin Y solution, porcine gelatine, ascorbic acid 2-phosphate (AA2P), Triton X-100, polydimethylsiloxane (PDMS, silicone), calcium chloride dihydrate, chlorotoxin, ethylenediaminetetraacetic acid (EDTA), glutaraldehyde (25%), hydrocortisone, insulin (human recombinant), l-glutamine, penicillin, streptomycin, trypsin EDTA, d-glucose, dimethyl sulphoxide (DMSO), AlamarBlue® resazurin sodium salt, F12-HAMS and Dulbecco's modified Eagle's medium (DMEM) were purchased from Sigma Aldrich (Poole, UK). Direct Red 80 (Sirius Red) was purchased from Fluka (Buchs, Switzerland). Chloroform, toluene, industrial methylated spirit (IMS), dichloromethane (DCM), and methanol were purchased from Fisher Scientific (Pittsburgh, PA, USA). The surfactant Hypermer B246-SO-M was received as a sample from Croda (Goole, UK). Collagenase A was purchased from Roche (Indianapolis, IN, USA). Human aortic endothelial cells (HAECs) and EC growth medium (EC GM) were purchased from PromoCell (Heidelberg, Germany). 4′,6-Diamidino-2-phenylindole (DAPI) solution and phalloidin tetramethylrhodamine (TRITC) were purchased from ThermoFisher Scientific (San Jose, CA, USA). Optimum cutting temperature tissue freezing medium (OCT-TFM) was purchased from Leica Biosystems (Newcastle, UK). Epidermal growth factor (EGF) was purchased from R&D systems (Minneapolis, MN, USA).

## Experimental

### Manufacturing of the PCL PolyHIPE scaffolds

#### Synthesis of PCL methacrylate

The PCL used in this study is 4-arm PCL methacrylate (4PCLMA). Throughout the paper, the term ‘PCL PolyHIPE’ will be used to describe 4PCLMA PolyHIPE unless otherwise stated. The following protocol was followed for the synthesis of PCLMA.

Under nitrogen flow, pentaerythritol (0.088 mol) and ε-caprolactone (0.353 mol) were mixed in a round flask at 160 °C while stirring continuously at 200 rpm. When the pentaerythritol was dissolved, 50 μL of tin(ii) 2-ethylhexanoate was added, and the system was removed from the oil bath to cool down. Synthesised PCL was dissolved in 300 mL of DCM, and then TEA (0.52 mol) was added. Reagents were stirred, and a further 200 mL of DCM was added to ensure everything was dissolved. The flask was placed in an ice bath. MAA (0.52 mol) was dissolved in 100 mL DCM and transferred into a dropping funnel. When the addition of MAA was completed, the ice bath was removed, and the system was kept at room temperature (RT) overnight with stirring at 375 rpm. To remove the TEA, MAA and salts formed, the methacrylated PCL was washed with HCl solution (1 M, 1000 mL) prepared from concentrated HCl (37%, 12 M) and deionised water and subsequently, the mixture was washed twice with deionised water. Nearly all solvent was evaporated using a rotary evaporator. PCLMA solution was transferred into a bottle filled with methanol and placed in a −80 °C freezer until the precipitate formed at the bottom (overnight). The supernatant methanol was removed, and fresh methanol was added. These steps were repeated at least three times. Any remaining solvent was removed by using the rotary evaporator. PCLMA was stored in an appropriate vessel in the freezer (−20 °C) for further use.

#### Characterization of 4PCL and 4PCLMA

To confirm the structure of 4PCL and 4PCLMA and also to measure the degree of methacrylation, proton (^1^H) nuclear magnetic resonance (NMR) spectroscopy analysis was performed on an AVANCE III spectrometer at 400 MHz. The spectra were recorded using an 8.2 kHz acquisition window, with 64k data points in 16 transients with a 60 s recycle delay (to ensure full relaxation). Deuterated chloroform was used as a diluent (CDCl_3_). Spectra were analyzed using the MestReNova software. Chemical shifts were referenced relative to CDCl_3_ at 7.26 ppm.

The weight average molecular weight (*M*_w_) and number average molecular weight (*M*_n_) distributions of 4PCLMA were determined using a Viscotek GPCmax VE200 gel permeation chromatography (GPC) system with a differential refractive index detector (Waters 410). Tetrahydrofuran was used as the eluting solvent at a flow rate of 1 mL min^−1^ at 40 °C, and polystyrene standards were used as the calibration sample.

#### Preparation of PCL HIPEs

4PCLMA (1.2 g) and the surfactant Hypermer B246 (10% w/w of polymer) were added into a glass vial and heated to 40 °C to dissolve the surfactant, which is crucial for emulsion stability. Solvent blend (150% w/w of polymer, 90% chloroform, 10% toluene (w/w)) and photoinitiator (10% w/w of polymer) were added in the 4PCLMA-surfactant mixture and mixed at 375 rpm using a magnetic stirrer for 1 minute at RT. Once the homogeneous mixture formed, 6 mL of water was added dropwise in 2 minutes and the emulsion was mixed for a further 2 minutes.

#### Manufacturing of PCL PolyHIPE scaffolds

PCL HIPEs were manufactured by polymerisation in silicone moulding and sectioning of 250 µm samples using a vibratome (Bio-Rad Polaron Division). For the fabrication of PCL PolyHIPE scaffolds, 250 µm sections of PCL PolyHIPE were used.

Briefly, PCL HIPE was pipetted into silicon templates and cured for 3 minutes on both sides using a UV curer with a 100 W cm^−2^ UV bulb (Omnicure Series 1000, Lumen Dynamics, Canada). The resulting parts were recovered, soaked in 100% methanol for 24 hours with four changes to remove any remaining contaminants of surfactant, solvent or uncured material. Then the samples were left in methanol (50% (v/v) in water) for 24 hours and water for a further 24 hours. Finally, the samples were taken out of the water and left in the freezer (−80 °C) for an hour then transferred into a vacuum oven and left for a day to preserve the porous structure of PCL PolyHIPE without any collapse. Scaffolds were cut into 5 mm circles using a biopsy punch (Stiefel, Slough, UK) for further experiments.

Air plasma (Diener Electronic, Ebhausen, Germany) was applied on both surfaces of the PCL PolyHIPE with a power of 50 W and a pressure of 0.8 mbar for 60 seconds to improve cell attachment to the hydrophobic surfaces, as demonstrated previously.^29,42^

#### Morphological characterisation of PCL PolyHIPE scaffolds

Scanning electron microscopy (SEM) was used to investigate the microarchitecture of the scaffolds. Samples were gold sputter-coated in 15 kV for 2.5 minutes to increase conductivity. A FEI Inspect F SEM (Philips/FEI XL-20 SEM, Cambridge, UK) was used with 10 kV power and 60 pores and 60 windows were selected randomly from 3 different areas of 3 different scaffolds, with the average pore and window sizes measured using ImageJ as described previously.^33^ A statistical correction factor (2/√3) was applied to micropore measurements to adjust the underestimation of the diameter because of uneven sectioning.^30,43^

#### Measurement of the liquid absorption capabilities of PCL PolyHIPE scaffolds

Liquid absorption capabilities of scaffolds were assessed by incubating weighed 5 mm circular scaffolds, either treated with air plasma or as non-treated, in 5 mL of PBS. Six samples for each group were measured. Scaffolds were incubated at 37 °C for 2 hours. At 10, 20, 30, 45, 60, and 120 minutes, they were blotted with filter paper to remove excess surface water and weighed. Percentage liquid absorption capabilities of the PCL PolyHIPE scaffolds were determined by using the following formula:(Weight_wet_ − weight_dry_)/weight_dry_ × 100

### Investigation of the effect of ascorbic acid 2-phosphate (AA2P) on the metabolic activity and collagen production of HDFs

#### Isolation of human dermal fibroblasts (HDFs)

All experiments were performed in accordance with the Guidelines of the National Research Ethics Service (NRES), and experiments were approved by the ethics committee at the Sheffield University Hospitals (REC ref., 15/YH/0177; REC opinion date, 03/06/2015). Informed consents were obtained from human participants of this study.

HDFs were isolated from skin grafts taken from patients using a well-established protocol.^44^ Briefly, the dermis was minced into 10 mm^2^ pieces and the pieces were incubated overnight at 37 °C in 0.5% (w/v) collagenase A solution. The cell suspension was then centrifuged at 1000 rpm (approximately 150*g*) for 5 minutes and resuspended and cultured in DMEM containing 10% (v/v) FBS, 100 IU mL^−1^ penicillin, 100 mg mL^−1^ streptomycin, 2 mM l-glutamine and 0.625 μg mL^−1^ amphotericin B. HDFs were used between passage 4–8. The investigations were carried out following the rules of the Declaration of Helsinki, 1975. Ethical approval for the tissue acquisition was granted by the National Research Ethics Service (NRES) Committee Yorkshire & The Humber–Sheffield (REC ref: 15/YH/0177, REC opinion date: 03/06/2015).

#### Measurement of metabolic activity and collagen production of HDFs

4 × 10^4^ HDFs were trypsinised, resuspended in 15 µl of DMEM, either supplemented with 50 µg mL^−1^ AA2P or non-supplemented and seeded to PCL PolyHIPE scaffolds and to 48-well plates. Cells were returned to an incubator (37 °C, 5% CO_2_) for 2 hours to allow cells to attach prior to adding 1 mL of growth medium. Scaffolds were transferred into a fresh well plate the next day, and the growth medium was refreshed every 2–3 days.

AlamarBlue® assay and Sirius Red staining were performed to investigate the effect of AA2P on the metabolic activity and collagen production of HDFs, respectively, over 21 days.^29^ Briefly, 0.1 mM AlamarBlue® working solution was prepared by 10× dilution of the 1 mM AlamarBlue® stock solution with growth medium. On days 1, 7, 14, and 21, growth media were removed, and the samples were washed with PBS. 1 mL of AlamarBlue® working solution was added to each well and incubated at 37 °C for 4 hours. After an incubation period, 200 µl of the solution was transferred into a 96-well plate, and the fluorescence readings were done at an excitation wavelength of 540 nm and an emission wavelength of 635 nm. Fresh samples were used for the measurements at each time point.

For collagen deposition, Sirius Red (direct 80) powder was dissolved in saturated picric acid (1 w/v%) to form Sirius Red solution (SRS) and filtered to ensure no particles remain. At days 1, 7, 14, and 21, growth media were removed, and the samples were washed with PBS. 1 mL of SRS was added to samples and incubated for 1 hour at room temperature (RT). SRS was removed, and the samples were washed every five minutes with deionized water and gentle orbital shaking until the water remained clear. They were submerged with 1 mL of 0.2 M sodium hydroxide (NaOH) : methanol (1 : 1) to destain and left for 30 minutes with gentle orbital shaking at RT. 150 μL of the destain solution in triplicate was transferred into a clear 96 well plate and read at an absorbance of 405 nm.

#### Preparation of the bio-functionalised PCL PolyHIPE scaffolds

**Fig. 1:**
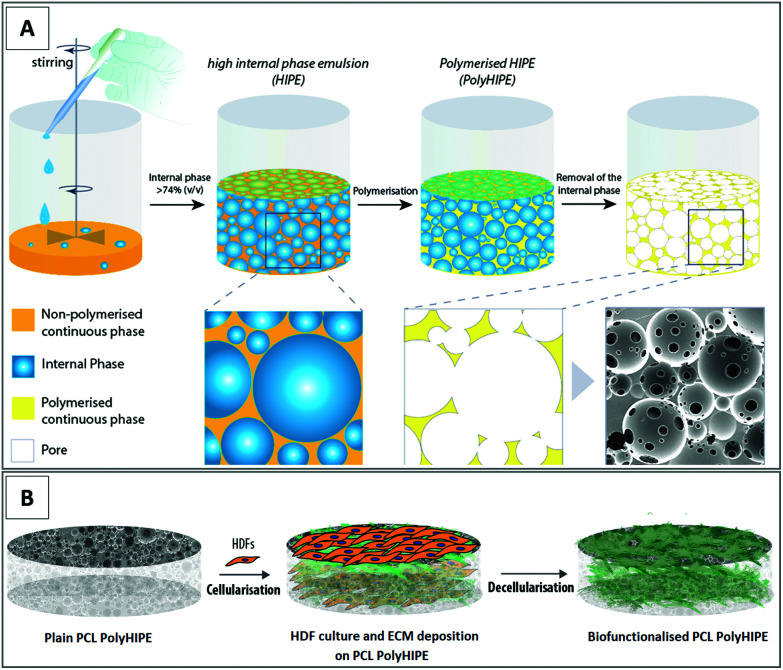
Fabrication and bio-functionalisation of the constructs. (A) Preparation of the emulsions made of photocurable PCL and water and polymerisation to form PCL PolyHIPE structure and (B) culture of HDFs and ECM deposition on PCL PolyHIPE scaffolds and decellularisation to prepare the bio-functionalised PCL PolyHIPE scaffolds.

Bio-functionalised scaffolds were prepared by cellularisation with human dermal fibroblasts (HDFs) and decellularisation of them after fibroblast-derived ECM decoration. Manufacturing routes of the PCL PolyHIPE (Fig. 1A) and the bio-functionalised PCL PolyHIPE scaffolds (Fig. 1B) are given in Fig. 1.

#### Cellularisation of PCL PolyHIPE scaffolds with HDF

PCL PolyHIPE scaffolds were washed with 100% ethanol four times (24 hours each) to remove any remaining contaminants of surfactant, solvent or uncured material. Then, they were left in 70% ethanol for 2 hours and then transferred into PBS in sterile conditions; four PBS washes were applied in 24 hours. 4 × 10^4^ HDFs were trypsinised, resuspended in 15 µl of DMEM and seeded to PCL PolyHIPE scaffolds (5 mm diameter), and the scaffolds were returned to an incubator (37 °C, 5% CO_2_) for 2 hours to allow cells to attach. After 2 hours, 1 mL of growth medium supplemented with 50 µg mL^−1^ AA2P was added to each well and incubated overnight. Scaffolds were transferred into a fresh well plate the next day, and AA2P supplemented DMEM was refreshed every 3 days. HDFs were cultured for 14 days on PCL PolyHIPE scaffolds under AA2P enriched conditions before decellularisation.

#### Decellularisation of the PCL PolyHIPE scaffolds cellularised with HDFs and the confirmation of DNA removal

Freezing and thawing (FT) + DNAse were used for the decellularisation of the scaffolds. Although FT has been found to be inefficient as a single-step method for the removal of native DNA,^45^ we have previously reported its efficiency when combined with DNAse.^33^ DNAse has also been reported as a safe method for the preservation of ECM while removing the genetic material.^46–48^

For decellularisation of the PCL PolyHIPE scaffolds, consecutive three FT cycles were applied to the scaffolds. For one FT cycle, scaffolds were left at −80 °C for 15 minutes and transferred into a 37 °C water bath for 30 minutes. Following the FT cycles, scaffolds were incubated in 1 mL of DNAse solution (0.2 mg mL^−1^) at 37 °C for 1 hour prior to washing with PBS.

A Quant-iT™ Picogreen® (PG) dsDNA kit (Invitrogen, Orlando, USA) was used to quantify the remaining DNA content and determine the effectiveness of the decellularisation protocol. Cellular and decellularised PCL PolyHIPE scaffolds (5 mm diameter) were washed with PBS three times and transferred into 24-well plates. 500 µl of cell digestion buffer (10 mM Tris-HCl, 1 mM ZnCl_2_ and 1% Triton-X100 in distilled water) was added onto the samples and incubated for 30 min at RT. Samples were then vortexed for 60 seconds and kept overnight at 4 °C. PG working solution was prepared by diluting 20× Tris–EDTA (TrE) 1 : 20 in dH_2_O (1× TrE) and diluting PG reagent 1 : 200 in 1× TrE. The scaffolds then underwent 3× FT cycles (10 min at −80 °C and 20 minutes at 37 °C, 15 seconds vortex between FT cycles). 100 µl of the lysate was mixed with 100 µl PG working solution in 96-well plates wrapped in foil and incubated at RT for 10 minutes. Fluorescence reading was done at an excitation wavelength of 485 nm and an emission wavelength of 528 nm. The efficiency of decellularisation was determined by comparing %DNA content in the cellular and decellularised scaffolds.^49–51^

The remaining collagen content after decellularisation was determined using Sirius Red staining as described in previous sections. Briefly, Sirius Red powder was dissolved in saturated picric acid (1% (w/v)) to form SRS and filtered to ensure no particles remained. Scaffolds were submerged in 1 mL of SRS and incubated for 1 hour. The solution was then removed, and scaffolds were washed every five minutes with distilled water while being mixed until the water remained clear. Scaffolds were submerged with a known volume of 5% perchloric acid or 0.2 M NaOH : methanol (1 : 1) to destain Sirius Red for 1 hour with gentle orbital shaking. 150 μL of the destain solution in triplicate was transferred into a clear 96 well plate and read at an absorbance of 405 nm.

In addition to quantitative methods, DNA removal and ECM preservation were also verified *via* fluorescent staining. Briefly, cellular and decellularised PCL PolyHIPE scaffolds were fixed in 3.7% formaldehyde (after 14 days of culture) and washed gently with PBS and submerged into 0.1% (v/v) Triton X 100 (in PBS) solution for 20 min. After serial PBS washes, phalloidin-TRITC (1 : 500 diluted in PBS from stock solution) solution was added onto samples to visualize F-actin and incubated for 30 min at RT in the dark. The samples were washed three times with PBS. In order to stain cell nuclei, 4′,6-diamidino-2-phenylindole (DAPI) solution (1 : 1000 diluted in PBS) was added onto the scaffolds and incubated for 10–15 min at RT in the dark, samples were then washed 3 times with PBS and directly imaged under a fluorescent microscope (Olympus IX3, Tokyo, Japan).

#### Mechanical characterisation of the scaffolds

Following the decellularisation, mechanical properties of the plain and decellularised dressings were measured using a uniaxial mechanical testing device. First, mechanical testing samples were cut into 20 mm × 5 mm pieces and clamped to the device with two tensile grips, and the tensile tests were performed on each sample using a Zwick Roell Z 0.5 mechanical testing machine equipped with a 500 N load cell as described previously.^30^ Grip distance and extension rate were set to 10 mm and 0.02 mm s^−1^, respectively. Both the force and elongation data were recorded. The Young's modulus was calculated using the linear-elastic region of each sample's stress–strain curves. The ultimate tensile strength (UTS) was calculated as that of the maximum force applied divided by the cross-sectional area of the sample. Elongation at yield is determined as the percentage elongation at the end of the linear-elastic region.

#### SEM of biological samples

Cellular and decellularised PCL PolyHIPE scaffolds at days 1, 7, 14, and 21 were washed three times with PBS after removing culture media and fixed in 2.5% (in PBS) glutaraldehyde at RT for 1 hour to preserve cell structure. They were rinsed with PBS for 15 minutes (3 times) and soaked in dH_2_O for 5 minutes. Following this, samples were subjected to serial ethanol washes to be dehydrated (35%, 60%, 80%, 90%, and 100% for 15 minutes for each concentration). Finally, samples were treated with drying agent HMDS/ethanol (1 : 1) for 1 hour and 100% HMDS for 5 minutes. Scaffolds were air-dried overnight in a fume hood and gold-coated at a current of 15 mA for 2.5 min with a gold sputter (Edwards sputter coater S150B, Crawley, England) prior to imaging under SEM (Philips/FEI XL-20 SEM; Cambridge, UK).

#### Evaluation of the efficiency of bio-functionalised PCL PolyHIPE scaffolds in terms of metabolic activity of human endothelial, fibroblast and keratinocyte cells

The bio-functionalised PCL PolyHIPE scaffolds were recellularised with three different cell types: (i) human aortic endothelial cells (HAECs), (ii) human dermal fibroblasts (HDFs), and (iii) human epidermal keratinocytes (HEKs), in order to evaluate the efficiency of them in terms of enhancing the metabolic activity as an indicator of wound healing *in vitro*.

#### Cellularisation of the bio-functionalised scaffolds with HDFs

HDFs were isolated and cultured as described in previous sections. Briefly, 4 × 10^4^ HDFs were trypsinised, resuspended in 15 µl of DMEM and seeded to either bio-functionalised or plain PCL PolyHIPE scaffolds (5 mm diameter), and the scaffolds were returned to an incubator (37 °C, 5% CO_2_) for 2 hours to allow cells to attach. After 2 hours, 1 mL of growth medium was added to each well and incubated overnight. Scaffolds were transferred into a fresh well plate the next day, and the growth medium was refreshed every 3 days. HDFs were cultured for 7 days on PCL PolyHIPE scaffolds, and the AlamarBlue assay was used to track the activity of HDFs on days 1, 4, and 7 on bio-functionalised and plain PCL PolyHIPE scaffolds as described in the previous sections.

#### Cellularisation of the bio-functionalised scaffolds with HEKs

HEKs were isolated from the skin, as described previously.^52^ Briefly, skin samples were cut into 0.5 cm^2^ pieces and incubated overnight in Difco-trypsin (0.1% (w/v) trypsin, 0.1% (w/v) d-glucose in PBS, pH 7.45) before being washed and maintained in PBS. Skin samples were transferred into a Petri dish and the epidermis was peeled off, the papillary surface of the epidermis was gently scraped, and basal keratinocytes were collected into the growth media. Cells were then harvested by centrifuging at 1000 rpm (approximately 150*g*) for 5 minutes, resuspended and seeded into 75 cm^2^ tissue culture flasks with the presence of a feeder layer (irradiated mouse 3T3 (i3T3) cells) and cultured in Green's media (66% DMEM (v/v), 21.6% F12-HAMS (v/v), 10% FCS (v/v), 0.5% insulin, 0.5% adenine, 0.1% T/T, 0.1% chlorotoxin, 0.016% hydrocortisone, 0.01% EGF, 100 IU ml^−1^ penicillin, 100 µg ml^−1^ streptomycin, 2 mM l-glutamine and 0.625 µg ml^−1^ amphotericin B).

The i3T3 feeder layer was removed first using 5 mL of 0.5 M sterile EDTA solution with 3–5 minutes incubation at 37 °C. After removal of the feeder layer, 4 × 10^4^ HEKs were trypsinised, resuspended in 15 µl of Green's media and seeded to either bio-functionalised or plain PCL PolyHIPE scaffolds (5 mm diameter). The scaffolds were returned to an incubator (37 °C, 5% CO_2_) for 2 hours to allow the cells to attach. After 2 hours, 1 mL of growth medium was added to each well and incubated overnight. Scaffolds were transferred into a fresh well plate the next day, and the growth medium was refreshed every 3 days. HEKs were cultured for 7 days on PCL PolyHIPE scaffolds, and the AlamarBlue assay was used to track the activity of HEKs on days 1, 4, and 7 on bio-functionalised and plain PCL PolyHIPE scaffolds as described in the previous sections.

#### Cellularisation of the bio-functionalised scaffolds with HAECs

HAECs (purchased from Promocell, Heidelberg, Germany) removed from liquid nitrogen were immediately thawed at 37 °C and transferred to a container with 10 mL of endothelial cell growth medium (EC GM). Cells were centrifuged, and the cell pellet was re-suspended in EC GM (supplemented with a total of 5% FBS and growth medium 2 kit supplements). The cell suspension was then transferred into 75 cm^2^ flasks and incubated at 37 °C, 5% CO_2_ (Thermo Scientific, Massachusetts, USA). The culture media was replaced every 3 days until they reached ∼80–90% confluency. HAECs were used between passages 2 and 6.

4 × 10^4^ HAECs were trypsinised, resuspended in 15 µl of EC GM and seeded to either bio-functionalised or plain PCL PolyHIPE scaffolds (5 mm diameter), and the scaffolds were returned to an incubator (37 °C, 5% CO_2_) for 2 hours to allow the cells to attach. After 2 hours, 1 mL of growth medium was added to each well and incubated overnight. Scaffolds were transferred into a fresh well plate the next day, and the growth medium was replenished every 3 days. HAECs were cultured for 7 days on PCL PolyHIPE scaffolds, and the AlamarBlue assay was used to track the activity of HAECs on days 1, 4, and 7 on bio-functionalised and plain PCL PolyHIPE scaffolds as described in the previous sections.

#### Haematoxylin and eosin (H&E) staining

Haematoxylin and eosin (H&E) staining of the PolyHIPE scaffolds has been performed as described previously.^30,53,54^ Briefly, on day 7, bio-functionalised and plain PCL PolyHIPE scaffolds cellularised with HDFs, HEKs, and HAECs were washed with PBS and fixed with 3.7% formaldehyde. Scaffolds were then transferred into cryomolds filled with freezing media and frozen. Sections with 6–10 μm thickness were taken on glass slides using the cryostat (Leica CM1860 UV, Milton Keynes, UK). Slides were stained with hematoxylin for 1.5 minutes and with eosin for 5 minutes. After washing with distilled water, slides were dehydrated in serial IMS washes and dunked into xylene. The slides were then mounted with DPX, and the images were captured using a light microscope (Motic BA210, China).

### Evaluation of the angiogenic potential of the bio-functionalised PCL PolyHIPE scaffolds using the *ex ovo* chick chorioallantoic membrane (CAM) assay

#### Implantation of the bio-functionalised and plain PCL PolyHIPE scaffolds to CAM

The detailed protocol of the CAM assay has previously been described elsewhere.^32,55^ In brief, fertilised chicken eggs (Gallus gallus domesticus) were purchased from Henry Stewart & Co. MedEggs (Norwich, UK) and cleaned with 20% industrial methylated spirit solution. Eggs were incubated at 37.5 °C for 3 days (until embryonic development day (EDD) 3) in a rocking egg incubator (RCOM King SURO, P&T Poultry, Powys, Wales). On EDD 3, the embryos were transferred gently into sterile Petri dishes and incubated at 38 °C in a cell culture incubator (Binder, Tuttlingen, Germany). The CAM assay was conducted with care using the guidelines of the Home Office, UK.

On EDD 7, bio-functionalised and plain PCL PolyHIPE scaffolds (5 mm diameter) were implanted onto the CAM. Images of the CAM area implanted with the scaffolds were acquired using a digital microscope, and the embryos were euthanised immediately after image acquisition on EDD 12.

#### Histological evaluation of the scaffolds on CAM

Hematoxylin and eosin (H&E) staining was performed as described previously.^31^ Briefly, scaffolds were cut together with a rim of surrounding CAM tissue and fixed in 3.7% formaldehyde, and the fixed samples were embedded in optimal cutting temperature freezing medium and frozen in liquid nitrogen for 3 minutes. Sections were cut 8–10 μm thick using a cryostat (Leica Biosystems, Nussloch, Germany) at −20 °C. Sections were then stained with hematoxylin for 90 seconds and eosin for 5 minutes. Finally, histological images were acquired under a light microscope (Motic BA210, China).

#### Fluorescent staining

For fluorescent staining, cellular and decellularised PCL PolyHIPE scaffolds on CAM were cut together with a rim of surrounding CAM tissue and fixed in 3.7% formaldehyde and washed gently with PBS. The phalloidin-TRITC and DAPI staining were performed as described in previous sections. Stained sections were directly imaged under a fluorescent microscope (Olympus IX3, Tokyo, Japan).

#### Quantification of angiogenesis

The angiogenic activity on CAM was quantified by using three different methods:

(i) From macro images by counting the blood vessels that converged towards the scaffolds as described previously.^56^ Briefly, all discernible blood vessels that are growing towards the scaffolds in a spoke wheel pattern within a 1 mm imaginary circle drawn around the scaffold were carefully counted. As described by Ribatti *et al.*, the blood vessels branching inside of the imaginary circle are counted as one blood vessel, whereas they are counted as two or more if the branching occurs outside the circle.^57^

(ii) From the processed macro images by calculating the total branch points as an indicator of formation of new blood vessels *via* sprouting angiogenesis.^54^ Briefly, a 12 × 12 mm^2^ area was cropped, and the color raw image was converted to greyscale for further analysis. To improve the discernability of the blood vessels, the following parameters were set to all images in ImageJ (Wayne Rasband, National Institutes of Health, USA); bandpass filter (filter large structures down to 40 pixels, filter small structures up to 3 pixels, and 5% tolerance of direction). Following the bandpass filter, all images were skeletonised using the find maxima command in Image J (prominence is set to 10 and output type is selected as segmented particles). Finally, the output skeletonised images were imported to AngioTool software (National Cancer Institute, USA) for quantifying the number of branch points.

(iii) From histological images by counting the blood vessels present.^54^ Briefly, all discernible blood vessels in the H&E-stained slides adjacent to the scaffolds were counted by two independent researchers using two independent microscopes at 10× magnification.

### Statistical analysis

Statistical analysis was carried out using one-way and two-way analysis of variance (ANOVA) using statistical analysis software (GraphPad Prism, California, USA). Where relevant, *n* values are given in figure captions. Error bars indicate standard deviations in the graphs unless otherwise stated.

## Results and discussion

### Synthesis and characterisation of 4PCL and 4PCLMA

**Fig. 2:**
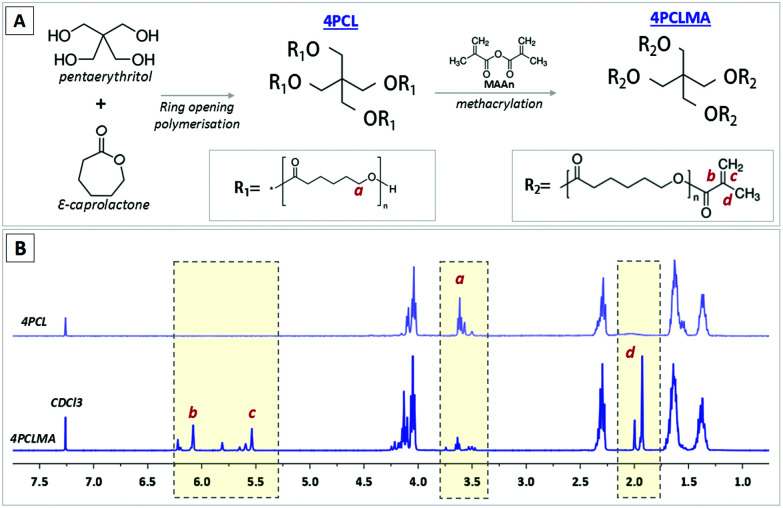
(A) Synthesis of 4PCL from the monomers *via* ring-opening polymerization and methacrylate functionalization of the hydroxyl end groups (4PCLMA), (B) chemical structure diagram and proton NMR spectrum of 4PCL and 4PCLMA and the relative assignments.

The chemical structure and ^1^H NMR spectra of 4PCL and 4PCLMA are given in Fig. 2. The peaks of the hydroxyl ends (–OH) are framed and labelled with “a”. These peaks represent the ends that were not methacrylated. The peaks of the methacrylate group are framed and labelled with “b”, “c” and “d”. These peaks only appear in the PCLMA and verify the metacrylation reaction. This ^1^H NMR data correlates well with the PCL methacrylate synthesized by Messori *et al.*^58^ GPC results showed that the *M*_w_ and *M*_n_ values were 2556 g mol^−1^ and 2214 g mol^−1^, respectively, and the polydispersity index (*M*_w_/*M*_n_, PDI) was calculated as 1.15.

### Fabrication and characterisation of the PCL PolyHIPE scaffolds

**Fig. 3:**
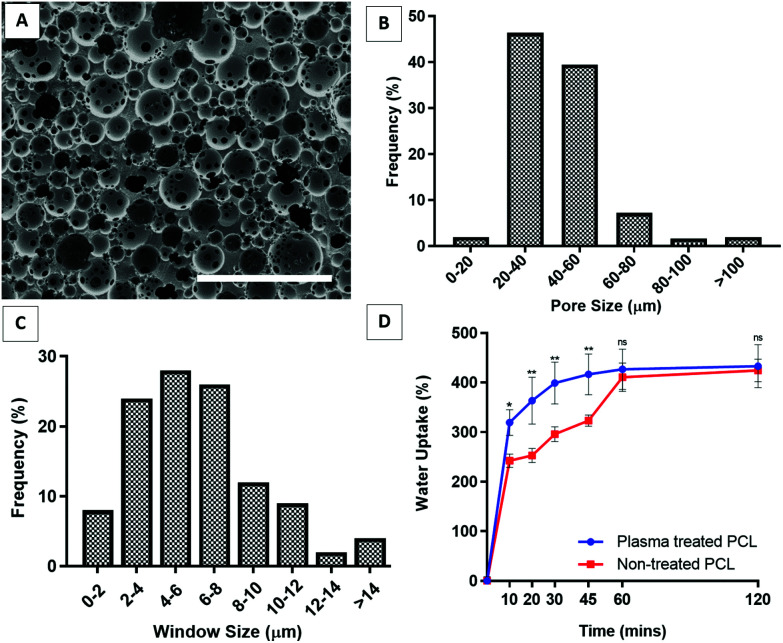
(A) The SEM images of the PCL PolyHIPE showing the microporous and interconnected structure of the constructs. (B) Pore size and (C) window size distribution of PCL PolyHIPE. (D) Liquid absorption capability of the plasma-treated and non-treated PCL PolyHIPE scaffolds. ** *p* < 0.01, * *p* < 0.05, ns not significant, *p* > 0.05, *n* = 3 ± SD. Scale bar represents 200 µm.

The images of the PCL PolyHIPE scaffolds showed that the scaffolds had a highly porous and open-cell morphology, characterised by the presence of windows on the walls of the pores (Fig. 3A). The pore sizes of the PCL PolyHIPE scaffold ranged from 6 to 110 µm; the average pore size (*D*) was 34.3 ± 11.8 µm, and 93% of the pores have pore sizes between 20–60 µm (Fig. 3B). The window sizes ranged from 2 to 22 µm, and the average window size (*d*) was 7.5 ± 3.6 µm (Fig. 3C), which gives the degree of interconnectivity (*d*/*D*) as 0.22 which is in line with the reported values in the literature.^30,42^ Similarly, in our previous study, the same solvent composition was used to dilute PCL, and the average pore size and the window size was found to be 34 ± 13 and 6 ± 2 µm, respectively.^29^ Pore size and interconnectivity are critical for the cell-scaffold interaction in terms of cell attachment, infiltration^59,60^ and vascularisation.^61,62^ Lordache *et al.* reported that a pore size range between 5 and 15 µm was enough to support fibroblast growth, and 20–125 µm was optimal for skin regeneration.^63^ Similarly, Yannas *et al.* reported that the minimum wound contraction time for the partial regeneration of adult mammalian skin was observed when collagen-glycosaminoglycan scaffolds with a pore size range of 20 ± 4 µm to 125 ± 35 µm were implanted into guinea pigs.^64^ For vascularisation, there is a range of different pore sizes that have been shown to support blood vessel ingrowth. Although the general consensus on the optimal pore size of vascularisation is to have pores of 100–300 µm, many reports demonstrated that a minimum of 50% porosity with approximately 30–100 µm pore size range is suitable for supporting new blood vessel formation within the scaffolds.^29,30,65–67^

Although PCL is one of the most widely used polymers in TE applications due to its advantages, such as being biocompatible, bioresorbable, and FDA approved as surgical sutures and for drug delivery applications,^26,30,40,68^ the main drawback of PCL is its hydrophobicity, which limits the cell–material interaction. To overcome this, surface plasma treatment is one of the most popular methods for enhancing cell attachment and growth.^42,69–73^ In our previous studies, we demonstrated that air plasma treatment changed the surface characteristics of polymers from hydrophobic to hydrophilic which consecutively improved cell attachment and cellular infiltration. Contact angles of the water droplets on non-treated and air plasma treated PCL PolyHIPEs were measured as 67 ± 4° and 96 ± 4°, respectively.^29,42^

The absorption capacity of wound dressings for wound exudate is a critical factor in their use.^9^ Our results showed that PCL PolyHIPE was able to take up approximately 400% as much liquid as its weight in less than 1 hour. Although plasma treatment enhanced the initial absorption capacity of the PCL PolyHIPE, it did not affect the total amount of absorbed liquid (Fig. 3D).

### The effect of AA2P on the metabolic activity and collagen production of HDFs on PCL PolyHIPE

**Fig. 4:**
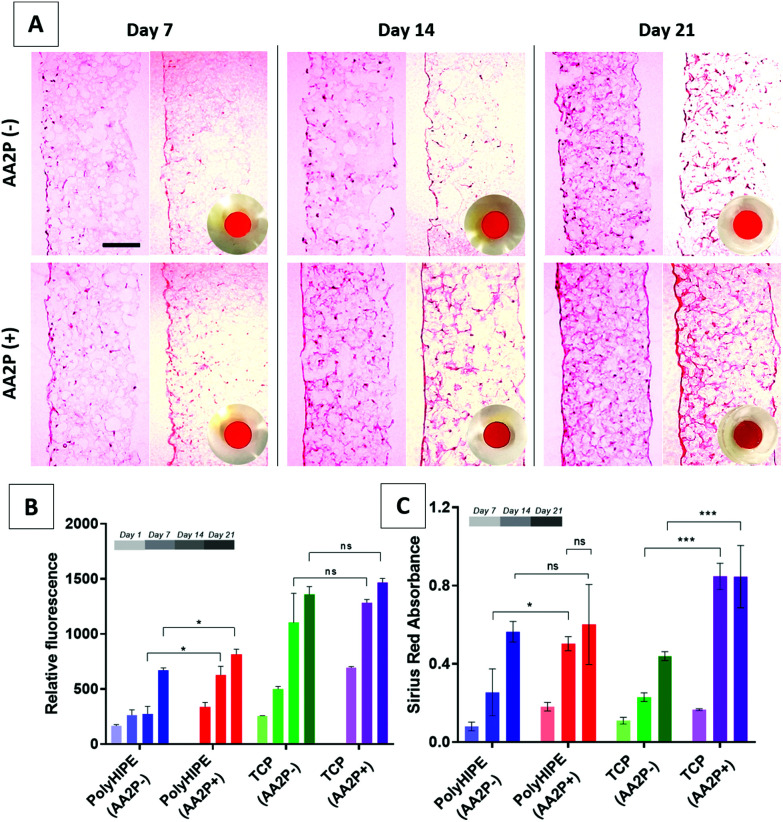
(A) H&E and Sirius Red-stained sections of the recellularised PCL PolyHIPE scaffolds with HDFs over 21 days. Circular images show the macro images of the Sirius Red-stained scaffolds. (B) AlamarBlue assay and (C) Sirius Red assay results showing cell viability and collagen production, respectively, over 21 days. AA2P was observed to increase both metabolic activity and collagen production compared with non-supplemented controls. Scale bars represent 100 µm. *** *p* < 0.001, * *p* < 0.05, ns not significant, *p* > 0.05, *n* = 3 ± SD.

AA2P was added to cell cultures to see if it could stimulate ECM production of cells on the scaffolds. The results of the AlamarBlue assay showed that the addition of AA2P to the growth medium significantly improved the metabolic activity of HDFs on PCL PolyHIPE on days 14 and 21, but not on day 7. Although it slightly increased the activity of HDFs growing on TCP at all time points, the difference was not statistically significant (Fig. 4B). The H&E-stained sections of the scaffolds also showed that cell density and localisation were higher within the scaffolds when the scaffolds were cultured in AA2P-supplemented media (Fig. 4A).

Similarly, supplementing AA2P significantly improved the collagen production of HDFs growing on PCL PolyHIPE scaffolds on day 14 but not on days 7 and 21 (Fig. 4C). No statistical difference was observed between days 14 and 21 in AA2P-supplemented groups. This data was confirmed by the Sirius Red staining of the sections. The results showed that AA2P significantly increased collagen production compared with the non-supplemented group (Fig. 4A).

**Fig. 5:**
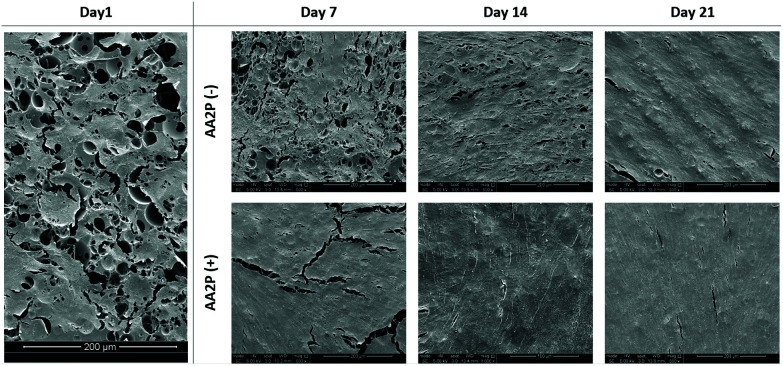
The SEM images of the HDFs cultured on PCL PolyHIPE scaffolds over 21 days either in the presence or absence of AA2P.

The SEM images of the HDFs cultured on PCL PolyHIPE scaffolds over 21 days either in the presence or absence of AA2P is given in Fig. 5. The images showed that in the AA2P-supplemented group, the surface was completely covered with HDFs after day 14, whereas in the non-supplemented group there were uncovered areas on day 14. The effect of AA2P on HDFs was more visible on day 7. The addition of AA2P significantly improved the coverage of the scaffold surface while the area covered by HDFs was lower in the control group.

Ascorbic acid plays an important role in the synthesis and modification of collagen.^74^ This is likely to be due to the fact that; (i) ascorbic acid acts as a co-factor for prolyl hydroxylase, an enzyme that is responsible for the cross-linking of collagen to form the triple helix structure,^75^ (ii) it stimulates collagen gene expression,^76^ (iii) it activates the gene transcription of collagen and stabilises the pro-collagen mRNA.^77,78^l-Ascorbic acid is the naturally occurring form and its use for increasing the collagen production of human fibroblasts dates back to the 1970s.^79^ However, ascorbic acid has been shown to have low chemical stability in culture.^80^ AA2P is the widely used form of ascorbic acid in cell culture applications and has been proven to be effective besides its stability.^81^

### Decellularisation of the PCL PolyHIPE scaffolds populated with HDFs

The overall aim of decellularisation is the successful removal of genetic material to avoid a possible immune response^82^ while preserving the ECM.^46^ Several decellularisation methods have been described in the literature, including chemical, enzymatic, and mechanical techniques. For decellularisation of the whole organs or tissues, it is desirable to combine multiple methods with longer washing steps.^46,83^ In contrast, for decellularisation of the *in vitro* generated ECM, less disruptive methods are preferable.^36,49,84–87^

**Fig. 6:**
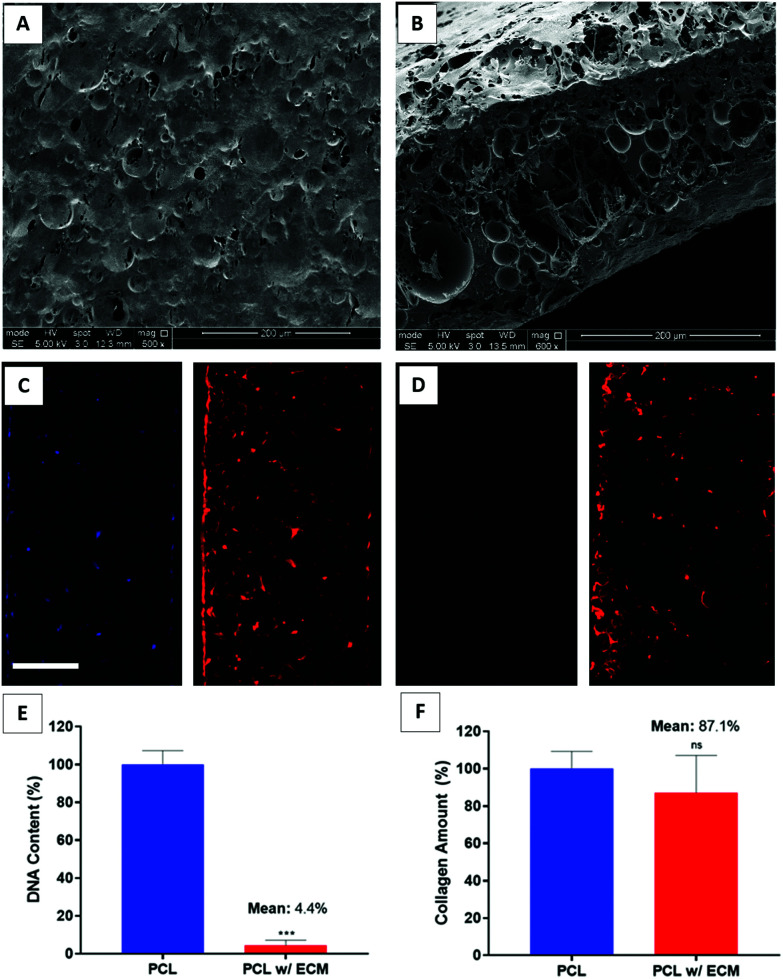
SEM images of the (A) surface and (B) cross-sections of the decellularised PCL PolyHIPE scaffolds. DAPI (blue) and phalloidin-TRITC (red) stained sections of the (C) cellular and (D) decellularised scaffolds. Graphs show the confirmation of (E) the DNA removal with Picogreen assay and (F) the preservation of the deposited collagen with Sirius Red assay. Scale bars represent 100 µm for fluorescent images. *** *p* < 0.001, ns not significant, *p* > 0.05, *n* = 3 ± SD.

In this study, we used FT + DNAse to decellularise our scaffolds. Decellularisation was confirmed by quantification of the DNA content and the verification of the removal of DNA in DAPI-stained sections. The remnant DNA remaining at the end of decellularisation was low enough to satisfy the suggested criteria for a successful decellularisation.^88^ The results demonstrated that FT + DNAse was effective in removing the genetic material up to 95.6% while preserving 87.1% of the deposited collagen onto PCL PolyHIPE (Fig. 6E and F). The removal of the DNA was further confirmed with DAPI/phalloidin stained sections (Fig. 6C and D). The SEM images of the decellularised scaffolds showed that the multiple steps of the decellularisation process seemed to disrupt the cell layer covering the surface of the scaffold and created a porous structure when compared with cellular scaffolds which were covered completely with HDFs after 14 days of culture (Fig. 6A and B). We have previously evaluated the efficiency of several decellularisation techniques and demonstrated that FT + DNAse is an effective method for the decellularisation of the scaffolds.^33^

### Mechanical characterisation of the bio-functionalised PCL PolyHIPE scaffolds

**Fig. 7:**
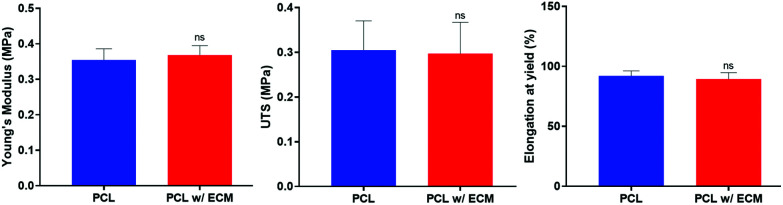
The mechanical testing results (Young's modulus, UTS, and elongation at yield) of plain and ECM containing PCL PolyHIPE scaffolds. ns not significant, *p* > 0.05, *n* = 3 ± SD.

No statistical difference was observed between PCL and PCL with ECM groups in terms of Young's modulus, UTS, and elongation at yield. The results showed that the decellularisation technique used in this study and the deposition of ECM onto the PCL PolyHIPE scaffolds did not alter the mechanical properties. The Young's moduli of the scaffolds were calculated as 0.35 ± 0.01 MPa and 0.37 ± 0.02 MPa for PCL and PCL with ECM scaffolds, respectively. The UTS of the scaffolds were 0.31 ± 0.03 MPa and 0.30 ± 0.03 MPa for PCL and PCL with ECM scaffolds, respectively. Finally, maximum elongations within the elastic region for PCL and PCL with ECM scaffolds were 92.10 ± 2.40% and 89.40 ± 3.10%, respectively. Very similar mechanical testing results have previously been measured for different applications of PCL PolyHIPE scaffolds with a similar composition.^29,30^ The mechanical testing results of plain and ECM containing PCL PolyHIPE scaffolds are summarised in Fig. 7.

### Investigation of bio-functionalised PCL PolyHIPE scaffolds on the metabolic activity of HDFs, HEKs and HAECs *in vitro*

Several types of cells, including neutrophils, macrophages, mast cells, T cells and B cells, take part in the inflammatory phase of wound healing.^89,90^ Following the inflammatory phase, fibroblasts play critical roles in the tissue repair phase^91,92^ and keratinocytes in the re-epithelialisation phase.^93^ Neovascularisation (endothelial cells) take important roles in inflammation and tissue repair phases for providing oxygen and nutrients, waste removal, and transmigration of inflammatory cells to the wound area.^12,13,15^ Considering the complex stages of wound healing, one approach is to investigate the metabolic activity and proliferation of cells that play a key role in wound healing to estimate the wound healing performance *in vitro*.^94,95^ Accordingly, we investigated the effect of *in vitro* generated fibroblast-derived ECM for improving the activity of fibroblasts, endothelial cells and keratinocyte cell types.

**Fig. 8:**
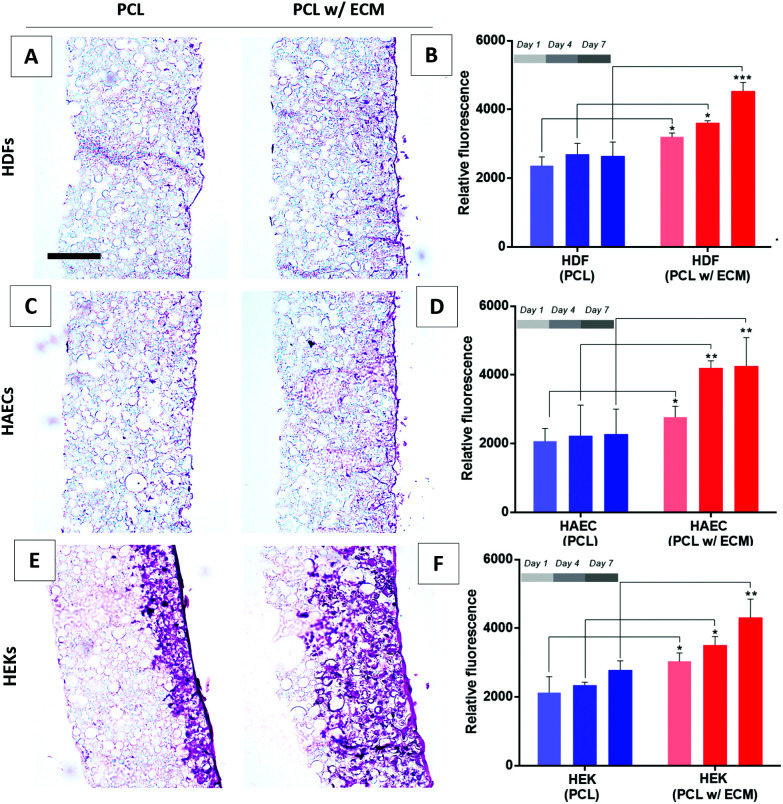
(A) H&E-stained sections of the HDFs cultured for 7 days on bio-functionalised scaffolds and (B) the metabolic activity of HDFs over 7 days. (C) H&E-stained sections of the HAECs cultured for 7 days on bio-functionalised scaffolds and (D) the metabolic activity of HAECs over 7 days. (E) H&E stained sections of the HEKs cultured for 7 days on bio-functionalised scaffolds and (F) the metabolic activity of HEKs over 7 days. Scale bars represent 100 µm. *** *p* < 0.001, ** *p* < 0.01, * *p* < 0.05, *n* = 3 ± SD.

AlamarBlue assay results showed that when HDFs, HAECs, and HEKs were cultured on plain PCL scaffolds there was no significant increase in their activities at any time point over 7 days. In contrast, all three types of cells showed an increase in their metabolic activities over 7 days when cultured on bio-functionalised PCL PolyHIPE scaffolds (Fig. 8B, D and F). This data was further confirmed with the H&E-stained sections of scaffolds cultured with HDFs (Fig. 8A), HAECs (Fig. 8C), and HEKs (Fig. 8E) at the end of 7-day culture. Although the histological images clearly showed the positive impact of the presence of *in vitro* generated ECM on their growth on the PCL PolyHIPE scaffolds for all three cells, the most significant difference was observed for HEKs where the proliferation and infiltration were dramatically higher in bio-functionalised scaffolds compared with the controls (Fig. 8E).

This increase in the attachment and growth of HDFs, HAECs, and HEKs when an *in vitro* fibroblast-derived matrix was present in the scaffolds was likely to be due to several reasons. Cell adhesion is modulated by integrins that can recognise ECM proteins.^96^ Besides the effect of better adhesion on the viability of cells, ECM proteins have also been reported to improve cell attachment and growth.^84,97,98^ Wong *et al.* have previously demonstrated that fibroblast-derived ECM contains laminin, collagen IV and fibronectin, alongside various other ECM proteins and some ECM associated proteins.^99^ Similarly, Castaldo *et al.* showed the presence of fibronectin, collagen, laminin, and tenascin in the composition of a fibroblast-derived matrix.^100^ In addition to the bioactive ECM components, the deposition of ECM onto the surfaces has been shown to increase the surface roughness which is likely to enhance the initial cell attachment.^101^

The increase in the metabolic activity of HAECs in the presence of fibroblast-derived ECM is likely to be due to fibroblasts playing a key role in endothelial cell regulation by producing significant amounts of ECM and pro-angiogenic factors which control the shape and density of blood vessels.^102,103^ Although fibroblasts can secrete pro-angiogenic factors, their main role is to form a rich extracellular environment in which endothelial cells can be embedded to form tubules.^104^ We have previously reported the positive impact of HDFs on the metabolic activity^31^ and monolayer formation of endothelial cells.^53^

The significant effect of the fibroblast-derived matrix on HEK metabolic activity and infiltration can be explained by the interplay between fibroblasts and keratinocytes in their natural environment.^91^ This has been widely studied, and the literature provides consistent evidence that keratinocytes show enhanced proliferation, differentiation, basement membrane deposition, and reduced apoptosis in the presence of fibroblasts.^105,106^ This cross-talk is mostly regulated *via* growth factors which may potentially be trapped in the ECM deposited by the HDFs. Fibroblasts have been shown to enhance the re-epithelialisation of the superficial incisional wounds created in human skin equivalents.^107^ In addition to the physical presence of fibroblasts, the presence of fibroblast-derived ECM has also been shown to support keratinocyte proliferation and control differentiation.^99^

### Investigation of the angiogenic potential of the bio-functionalised PCL PolyHIPE scaffolds in *ex ovo* CAM assay

The CAM assay is a well-established protocol, not only for the assessment of angiogenic potential, but also for the initial *in vivo* response to biomaterials.^29,31,32,53,55,56^

Our group, alongside others, has been trying to reduce the number of higher-order animals used in animal experiments during pre-clinical trials. Towards this aim, we adapted a chick chorioallantoic membrane (CAM) assay for testing pro-angiogenic biomaterials prior to detailed pre-clinical tests.

The CAM assay is a very well-known angiogenesis model that has been approved by the United States Food and Drug Administration (FDA) in 2006 to be used in the development of products for chronic cutaneous ulcer and burn wounds with a guidance for industry.^108^ In this guidance, it is stated that the stimulation of angiogenesis associated with wound healing can be tested using the CAM model (article 3, subparagraph A, animal wound models). With this guideline, the CAM model has been approved as one of the pre-clinical tests for the production of therapeutic products for chronic and burn wounds and has taken an important step towards expanding its use both in industry and research.^109^

The current direction in animal research is focused on reducing the use of animals, improving the well-being of laboratory animals used, and producing high-quality science even when these conditions are met. Accordingly, the CAM assay is one of the most supportive models of the 3R (replace, reduce, refine) approach introduced by Russel & Burch^110^ because CAM is a membrane that dynamically produces new blood vessels in response to biomaterials and drugs, and it is not innervated in the first 14 days of embryo development. The American institutional animal care and use board (IACUC)^111^ and the National Institutes of Health (NIH)^112^ stated that a chick embryo that has not reached the 15th day of the development period will not experience pain and therefore there are no ethical restrictions or pre-protocol. CAM is easily accessible for experimental interventions as it remains above the developing embryo (especially the *ex ovo* version of the CAM assay). Thus, being able to study *in vivo* angiogenesis without being in direct contact with the embryo makes it ethically more acceptable than many animal models, and it is the main reason why the FDA supports the use of this model by researchers before detailed animal experiments.

The *ex ovo* CAM assay results demonstrated that bio-functionalised PCL PolyHIPE scaffolds stimulated angiogenesis over 5 days. None of the implanted scaffolds affected the embryo survival rate, which was over 75% for all groups. In our previous protocol paper, we reported the average survival rates of the *ex ovo* (shell-less) cultured chick embryos as 25%, 68%, and 83% by inexperienced, intermediate, and experienced users, respectively.^55^

**Fig. 9:**
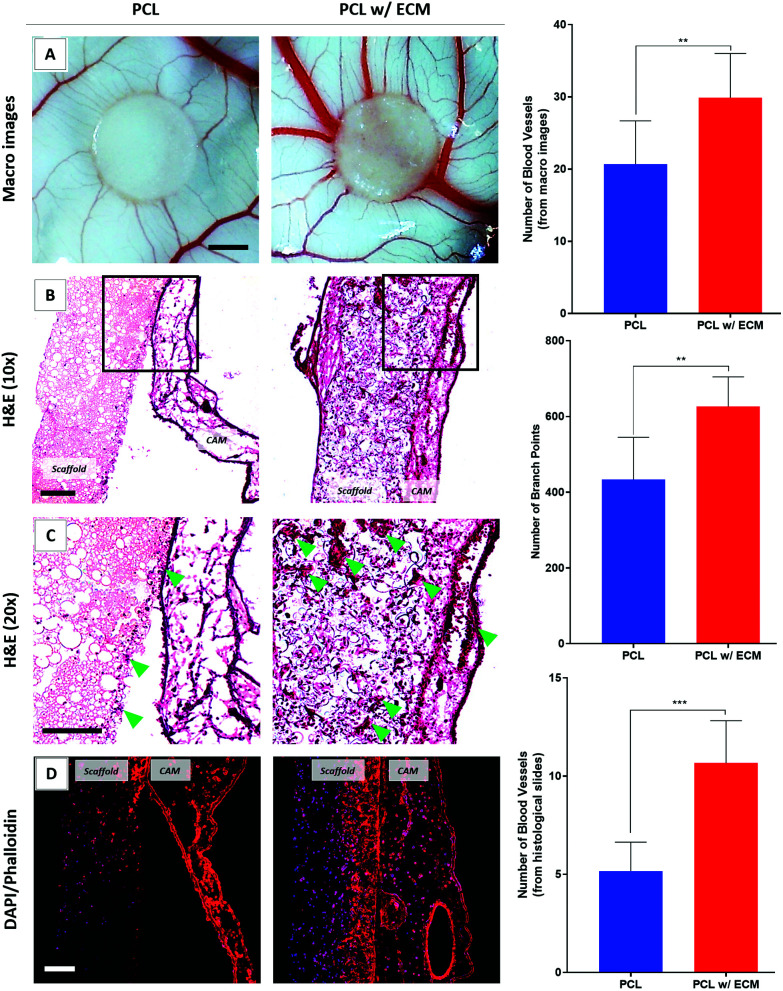
Evaluation of the angiogenic performance of the plain PCL and bio-functionalised scaffolds. (A) Macro images, (B) lower and (C) higher magnifications of the H&E-stained sections. Blood vessels are shown with green arrows. (D) Phalloidin-TRITC (red)/DAPI (blue) stained sections of the scaffolds. Graphs show the quantified results. Scale bars represent 2 mm for macro images of the CAM (A) and 100 μm for H&E (B and C) and fluorescent stained (D) sections. ***p < 0.001, **p < 0.01, *n* = 3 ± SD.

Assessment of blood vessel formation on the CAM showed that the inclusion of ECM had a dramatic effect on the number of discernible blood vessels and branching points (Fig. 9A). Blood vessels observed in the histological slides went up by a factor of 2.1 in the presence of *in vitro* generated ECM on the scaffolds compared with plain controls (Fig. 9B and C).

As well as the angiogenic performance of the bio-functionalised scaffolds, evaluation of the H&E and fluorescent stained sections of the scaffolds on CAM revealed that tissue integration to bio-functionalised scaffolds was significantly higher when compared with controls. In contrast, as can be seen in the H&E and fluorescent stained sections, there was limited tissue infiltration into plain PCL PolyHIPE which was loosely attached to the CAMs and peeled off from the surface of the CAM after the termination of the assay (Fig. 9D).

Fibroblasts have previously been demonstrated to have important roles in the angiogenic process by generating ECM molecules such as collagen and fibronectin,^113,114^ growth factors, and pro-angiogenic factors.^102,103^ Several ECM molecules, including collagen, fibronectin, and laminin, have been shown to possess important characteristics for angiogenesis and wound healing.^115,116^ All these three ECM molecules have been reported to be present in fibroblast-derived ECM.^99,100^ Alongside the ECM components that have the potential to promote angiogenesis, the fibroblast-derived matrix has also been shown to store pro-angiogenic factors such as VEGF,^117,118^ bFGF and hepatocyte growth factor (HGF).^119^ In addition, *in vitro* generated ECM has previously been shown to promote the vascularisation of the implanted constructs in a rat animal model^85^ and in a CAM assay,^33^ and the authors hypothesised that increased angiogenic activity was due to the bioactive ECM components and angiogenic factors that are stored in the generated ECM.

## Conclusions

In this study, bio-functionalised constructs were manufactured successfully by decellularisation of 14-day cultured HDFs on PCL PolyHIPE scaffolds. The decoration of PCL PolyHIPE scaffolds with fibroblast-derived ECM demonstrated an effective way not to enhance the metabolic activities of three major cell types that play a critical role in wound healing, but also markedly induce angiogenesis in *ex ovo* CAM assays. This proof-of-concept study provided a unique methodology to combine the advantages of a PCL PolyHIPE, including high tunability of mechanical and physical properties and ease of processing, with the biological complexity of fibroblast ECM to stimulate angiogenesis and help drive wound healing. The use of cells to deposit their own matrix resolves the need for the addition of each and every biologically active component exogenously. Finally, when looked at from a clinical point of view, using a patient's own cells prior to wound management removes any risk of disease transmission and avoids the risk of immune rejection.

In conclusion, fibroblast-ECM functionalised PCL PolyHIPE scaffolds appear to have great potential to be used as an active wound dressing to promote angiogenesis and accelerate wound healing. This study can easily be adapted for its use in patient-specific wound healing applications with the use of autologous fibroblasts isolated from the patient. Given the successful outcome of the detailed analysis of our scaffolds on the CAM bioassay, we are confident that we can proceed to further animal trials of the ECM-decorated PCL PolyHIPE scaffolds in our follow-up studies.

## Ethical statement

All experiments were performed in accordance with the Guidelines of the National Research Ethics Service (NRES), and experiments were approved by the ethics committee at the Sheffield University Hospitals (REC ref., 15/YH/0177; REC opinion date, 03/06/2015). Informed consents were obtained from human participants of this study.

The authors state that they have obtained appropriate institutional review board approval or have followed the principles outlined in the Declaration of Helsinki for all human or animal experimental investigations. All CAM assay experiments were carried out considering the 3R (replace, reduce, refine) approach and according to Home Office, UK, guidelines. The authors gratefully thank Dr Sandra van Meurs for her help on NMR analysis and interpretation.

## Author contributions

SD contributed to the experimental design, analysis, data acquisition, and interpretation of data, statistical analysis, and drafting of the manuscript. BAD contributed to the experimental design, analysis, data acquisition, interpretation of data and drafting of the manuscript. FC and SM contributed with their supervision and critical revision and editing of the manuscript.

## Conflicts of interest

There are no conflicts to declare.

## Supplementary Material

BM-009-D1BM01262B-s001

## References

[cit1] Lee S. H., Jeong S. K., Ahn S. K. (2006). Yonsei Med. J..

[cit2] Forslind B., Engström S., Engblom J., Norlén L. (1997). J. Dermatol. Sci..

[cit3] Rees J. (1999). Lancet.

[cit4] Dong R. H., Jia Y. X., Qin C. C., Zhan L., Yan X., Cui L., Zhou Y., Jiang X., Long Y. Z. (2016). Nanoscale.

[cit5] Stroncek J. D. and Reichert W. M., in Indwelling Neural Implants: Strategies for Contending with the in Vivo Environment, 2007, pp. 3–38 21204399

[cit6] Kawasumi A., Sagawa N., Hayashi S., Yokoyama H., Tamura K. (2013). Curr. Top. Microbiol. Immunol..

[cit7] Boateng J., Catanzano O. (2015). J. Pharm. Sci..

[cit8] Simões D., Miguel S. P., Ribeiro M. P., Coutinho P., Mendonça A. G., Correia I. J. (2018). Eur. J. Pharm. Biopharm..

[cit9] Andleeb A., Dikici S., Waris T. S., Bashir M. M., Akhter S., Chaudhry A. A., MacNeil S., Yar M. (2020). J. Tissue Eng. Regener. Med..

[cit10] Azam M., Dikici S., Roman S., Mehmood A., Chaudhry A. A., Rehman I. U., MacNeil S., Yar M. (2019). J. Biomater. Appl..

[cit11] Mele E. (2016). J. Mater. Chem. B.

[cit12] Pierce G. F., Vande Berg J., Rudolph R., Tarpley J., Mustoe T. A. (1991). Am. J. Pathol..

[cit13] Takeshita S., Zheng L. P., Brogi E., Kearney M., Pu L. Q., Bunting S., Ferrara N., Symes J. F., Isner J. M. (1994). J. Clin. Invest..

[cit14] Grazul-Bilska A. T., Johnson M. L., Bilski J. J., Redmer D. A., Reynolds L. P., Abdullah A., Abdullah K. M. (2003). Drugs Today.

[cit15] Velnar T., Bailey T., Smrkolj V. (2009). J. Int. Med. Res..

[cit16] Tonnesen M. G., Feng X., Clark R. A. F. (2000). J. Invest. Dermatol. Symp. Proc..

[cit17] Ignatova M., Manolova N., Markova N., Rashkov I. (2009). Macromol. Biosci..

[cit18] Toncheva A., Paneva D., Manolova N., Rashkov I. (2011). Macromol. Res..

[cit19] Amini F., Semnani D., Karbasi S., Banitaba S. N. (2019). Int. J. Polym. Mater. Polym. Biomater..

[cit20] Francis L., Meng D., Locke I. C., Knowles J. C., Mordan N., Salih V., Boccaccini A. R., Roy I. (2016). Polym. Int..

[cit21] Jung S. M., Yoon G. H., Lee H. C., Shin H. S. (2015). J. Biomater. Sci.,
Polym. Ed..

[cit22] Croisier F., Atanasova G., Poumay Y., Jérôme C. (2014). Adv. Healthcare Mater..

[cit23] Augustine R., Kalarikkal N., Thomas S. (2016). Appl. Nanosci..

[cit24] Abdelgawad A. M., Hudson S. M., Rojas O. J. (2014). Carbohydr. Polym..

[cit25] Zhang Y., Jiang M., Zhang Y., Cao Q., Wang X., Han Y., Sun G., Li Y., Zhou J. (2019). Mater. Sci. Eng., C.

[cit26] Woodruff M. A., Hutmacher D. W. (2010). Prog. Polym. Sci..

[cit27] Cheng Z., Teoh S. H. (2004). Biomaterials.

[cit28] Miroshnichenko S., Timofeeva V., Permyakova E., Ershov S., Kiryukhantsev-Korneev P., Dvořaková E., Shtansky D., Zajíčková L., Solovieva A., Manakhov A. (2019). Nanomaterials.

[cit29] Aldemir Dikici B., Dikici S., Reilly G. C., MacNeil S., Claeyssens F. (2019). Materials.

[cit30] Aldemir Dikici B., Sherborne C., Reilly G. C., Claeyssens F. (2019). Polymer.

[cit31] Dikici S., Claeyssens F., MacNeil S. (2020). Tissue Eng. Regener. Med..

[cit32] Dikici S., Claeyssens F., MacNeil S. (2019). J. Biomater. Appl..

[cit33] Aldemir Dikici B., Reilly G. C., Claeyssens F. (2020). ACS Appl. Mater. Interfaces.

[cit34] Kim B. S., Kim H., Gao G., Jang J., Cho D. W. (2017). Biofabrication.

[cit35] Datta N., Pham Q. P., Sharma U., Sikavitsas V. I., Jansen J. A., Mikos A. G. (2006). Proc. Natl. Acad. Sci. U. S. A..

[cit36] Pham Q. P., Kurtis Kasper F., Scott Baggett L., Raphael R. M., Jansen J. A., Mikos A. G. (2008). Biomaterials.

[cit37] Das S., Jang J. (2018). J. 3D Print. Med..

[cit38] Hoshiba T., Chen G., Endo C., Maruyama H., Wakui M., Nemoto E., Kawazoe N., Tanaka M. (2016). Stem Cells Int..

[cit39] Pati F., Jang J., Ha D. H., Won Kim S., Rhie J. W., Shim J. H., Kim D. H., Cho D. W. (2014). Nat. Commun..

[cit40] Aldemir Dikici B., Claeyssens F. (2020). Front. Bioeng. Biotechnol..

[cit41] Desjardins-Park H. E., Foster D. S., Longaker M. T. (2018). Regener. Med..

[cit42] Owen R., Sherborne C., Paterson T., Green N. H., Reilly G. C., Claeyssens F. (2016). J. Mech. Behav. Biomed. Mater..

[cit43] Barbetta A., Cameron N. R. (2004). Macromolecules.

[cit44] Bullock A. J., Higham M. C., MacNeil S. (2006). Tissue Eng..

[cit45] Lu H., Hoshiba T., Kawazoe N., Chen G. (2012). J. Biomed. Mater. Res., Part A.

[cit46] Gilpin A., Yang Y. (2017). BioMed Res. Int..

[cit47] Crapo P. M., Gilbert T. W., Badylak S. F. (2011). Biomaterials.

[cit48] Perea-Gil I., Uriarte J. J., Prat-Vidal C., Gálvez-Montón C., Roura S., Llucià-Valldeperas A., Soler-Botija C., Farré R., Navajas D., Bayes-Genis A. (2015). Am. J. Transl. Res..

[cit49] Sadr N., Pippenger B. E., Scherberich A., Wendt D., Mantero S., Martin I., Papadimitropoulos A. (2012). Biomaterials.

[cit50] Caralt M., Uzarski J. S., Iacob S., Obergfell K. P., Berg N., Bijonowski B. M., Kiefer K. M., Ward H. H., Wandinger-Ness A., Miller W. M., Zhang Z. J., Abecassis M. M., Wertheim J. A. (2012). Am. J. Transplant..

[cit51] Pereira R. H. A., Prado A. R., Del Caro L. F. C., Zanardo T. É. C., Alencar A. P., Nogueira B. V. (2019). Sci. Rep..

[cit52] Ghosh M. M., Boyce S., Layton C., Freedlander E., Mac Neil S. (1997). Ann. Plast. Surg..

[cit53] Dikici S., Claeyssens F., MacNeil S. (2020). ACS Biomater. Sci. Eng..

[cit54] Dikici S., Mangir N., Claeyssens F., Yar M., MacNeil S. (2019). Regener. Med..

[cit55] Mangir N., Dikici S., Claeyssens F., MacNeil S. (2019). ACS Biomater. Sci. Eng..

[cit56] Dikici S., Bullock A. J., Yar M., Claeyssens F., MacNeil S. (2020). Microvasc. Res..

[cit57] Ribatti D., Nico B., Vacca A., Presta M. (2006). Nat. Protoc..

[cit58] Messori M., Degli Esposti M., Paderni K., Pandini S., Passera S., Riccò T., Toselli M. (2013). J. Mater. Sci..

[cit59] Murphy C. M., Haugh M. G., O'Brien F. J. (2010). Biomaterials.

[cit60] Zhang Y., Fan W., Ma Z., Wu C., Fang W., Liu G., Xiao Y. (2010). Acta Biomater..

[cit61] Karageorgiou V., Kaplan D. (2005). Biomaterials.

[cit62] Klenke F. M., Liu Y., Yuan H., Hunziker E. B., Siebenrock K. A., Hofstetter W. (2008). J. Biomed. Mater. Res., Part A.

[cit63] Iordache F., in Materials for Biomedical Engineering, 2019, pp. 35–60

[cit64] Yannas I. V., Lee E., Orgill D. P., Skrabut E. M., Murphy G. F. (1989). Proc. Natl. Acad. Sci. U. S. A..

[cit65] Oliviero O., Ventre M., Netti P. A. (2012). Acta Biomater..

[cit66] Hong J. K., Bang J. Y., Xu G., Lee J. H., Kim Y. J., Lee H. J., Kim H. S., Kwon S. M. (2015). Int. J. Nanomed..

[cit67] Mastrullo V., Cathery W., Velliou E., Madeddu P., Campagnolo P. (2020). Front. Bioeng. Biotechnol..

[cit68] Dikici S., Aldemir Dikici B., Bhaloo S. I., Balcells M., Edelman E. R., MacNeil S., Reilly G. C., Sherborne C., Claeyssens F. (2019). Front. Bioeng. Biotechnol..

[cit69] Ma Z., He W., Yong T., Ramakrishna S. (2005). Tissue Eng..

[cit70] Prabhakaran M. P., Venugopal J., Chan C. K., Ramakrishna S. (2008). Nanotechnology.

[cit71] Fujihara K., Kotaki M., Ramakrishna S. (2005). Biomaterials.

[cit72] Zander N. E., Orlicki J. A., Rawlett A. M., Beebe T. P. (2012). ACS Appl. Mater. Interfaces.

[cit73] Can-Herrera L. A., Ávila-Ortega A., de la Rosa-García S., Oliva A. I., Cauich-Rodríguez J. V., Cervantes-Uc J. M. (2016). Eur. Polym. J..

[cit74] Mangir N., Bullock A. J., Roman S., Osman N., Chapple C., MacNeil S. (2016). Acta Biomater..

[cit75] Prockop D. J., Kivirikko K. I. (1995). Annu. Rev. Biochem..

[cit76] Houglum K. P., Brenner D. A., Chojkier M. (1991). Am. J. Clin. Nutr..

[cit77] Kurata S. I., Senoo H., Hata R. I. (1993). Exp. Cell Res..

[cit78] Kishimoto Y., Saito N., Kurita K., Shimokado K., Maruyama N., Ishigami A. (2013). Biochem. Biophys. Res. Commun..

[cit79] Levene C. I., Bates C. J. (1970). J. Cell Sci..

[cit80] Du J., Cullen J. J., Buettner G. R. (2012). Biochim. Biophys. Acta, Rev. Cancer.

[cit81] Hata R.-I., Senoo H. (1989). J. Cell. Physiol..

[cit82] Akbari-Birgani S., Birgani M. T., Ansari H. (2019). Stem Cells Biomater. Regener. Med..

[cit83] Zilic L., Wilshaw S. P., Haycock J. W. (2016). Biotechnol. Bioeng..

[cit84] Datta N., Holtorf H. L., Sikavitsas V. I., Jansen J. A., Mikos A. G. (2005). Biomaterials.

[cit85] Pham Q. P., Kasper F. K., Mistry A. S., Sharma U., Yasko A. W., Jansen J. A., Mikos A. G. (2009). J. Biomed. Mater. Res., Part A.

[cit86] Thibault R. A., Scott Baggett L., Mikos A. G., Kasper F. K. (2010). Tissue Eng., Part A.

[cit87] Tour G., Wendel M., Tcacencu I. (2011). Tissue Eng., Part A.

[cit88] Syed O., Walters N. J., Day R. M., Kim H. W., Knowles J. C. (2014). Acta Biomater..

[cit89] Noble P. W., Jiang D. (2006). Proc. Am. Thorac. Soc..

[cit90] Jameson J., Ugarte K., Chen N., Yachi P., Fuchs E., Boismenu R., Havran W. L. (2002). Science.

[cit91] Werner S., Krieg T., Smola H. (2007). J. Invest. Dermatol..

[cit92] Halloran C. M., Slavin J. P. (2002). Surgery.

[cit93] Kirfel G., Herzog V. (2004). Protoplasma.

[cit94] Harishkumar M., Masatoshi Y., Hiroshi S., Tsuyomu I., Masugi M. (2013). BioMed Res. Int..

[cit95] Governa P., Carullo G., Biagi M., Rago V., Aiello F. (2019). Antioxidants.

[cit96] Hidalgo-Bastida L. A., Cartmell S. H. (2010). Tissue Eng., Part B.

[cit97] Shekaran A., García A. J. (2011). J. Biomed. Mater. Res., Part A.

[cit98] Shin H., Zygourakis K., Farach-Carson M. C., Yaszemski M. J., Mikos A. G. (2004). Biomaterials.

[cit99] Wong C. W., LeGrand C. F., Kinnear B. F., Sobota R. M., Ramalingam R., Dye D. E., Raghunath M., Lane E. B., Coombe D. R. (2019). Sci. Rep..

[cit100] Castaldo C., Di Meglio F., Miraglia R., Sacco A. M., Romano V., Bancone C., Della Corte A., Montagnani S., Nurzynska D. (2013). BioMed Res. Int..

[cit101] Baroncelli M., Van Der Eerden B. C. J., Chatterji S., Rull Trinidad E., Kan Y. Y., Koedam M., Van Hengel I. A. J., Alves R. D. A. M., Fratila-Apachitei L. E., Demmers J. A. A., Van De Peppel J., Van Leeuwen J. P. T. M. (2018). Tissue Eng., Part A.

[cit102] Black a. F., Berthod F., L'heureux N., Germain L., Auger F. a. (1998). FASEB J..

[cit103] Hudon V., Berthod F., Black A. F., Damour O., Germain L., Auger F. A. (2003). Br. J. Dermatol..

[cit104] Newman A. C., Nakatsu M. N., Chou W., Gershon P. D., Hughes C. C. W. (2011). Mol. Biol. Cell.

[cit105] Russo B., Brembilla N. C., Chizzolini C. (2020). Front. Immunol..

[cit106] Smith A., Huang M., Watkins T., Burguin F., Baskin J., Garlick J. A. (2020). J. Tissue Eng. Regener. Med..

[cit107] El Ghalbzouri A., Hensbergen P., Gibbs S., Kempenaar J., Van Der Schors R., Ponec M. (2004). Lab. Investig..

[cit108] U.S. Department of Health and Human Services Food and Drug Administration, 2006, https://www.fda.gov/media/71278/download

[cit109] Ribatti D. (2017). Reprod. Toxicol..

[cit110] Russell W. and Burch R., The principles of humane experimental technique, Methuen & Co., London, 1959

[cit111] Institutional Animal Care and Use Committee (IACUC), Brown Univ. Institutional Anim. Care Use Comm., 2019, 1 3, https://www.brown.edu/research/sites/research/files/policies/Avian%20Embryo%20Use%20Policy%2011Jan19.pdf

[cit112] Institute for Laboratory Animal and Research (1991). ILAR News.

[cit113] Sorrell J. M., Baber M. A., Caplan A. I. (2007). Cells Tissues Organs.

[cit114] Bishop E. T., Bell G. T., Bloor S., Broom I. J., Hendry N. F., Wheatley D. N. (1999). Angiogenesis.

[cit115] Macri L., Clark R. A. F. (2009). Skin Pharmacol. Physiol..

[cit116] Olczyk P., Mencner Ł., Komosinska-Vassev K. (2014). BioMed Res. Int..

[cit117] Griffith C. K., George S. C. (2009). Tissue Eng., Part A.

[cit118] Ito T. K., Ishii G., Chiba H., Ochiai A. (2007). Oncogene.

[cit119] Du P., Suhaeri M., Ha S. S., Oh S. J., Kim S. H., Park K. (2017). Acta Biomater..

