# Supplementary material for: Decellularised extracellular matrix decorated PCL PolyHIPE scaffolds for enhanced cellular activity, integration and angiogenesis
Source: Biomater Sci. 2021 Sep 23;9(21):7297–310. doi: 10.1039/d1bm01262b (PMC8547328; doi:10.1039/d1bm01262b)
Supplement: BM-009-D1BM01262B-s001 [file BM-009-D1BM01262B-s001.pdf]

Electronic Supplementary Material (ESI) for Biomaterials Science.
This journal is © The Royal Society of Chemistry 2021

Supplementary Figures

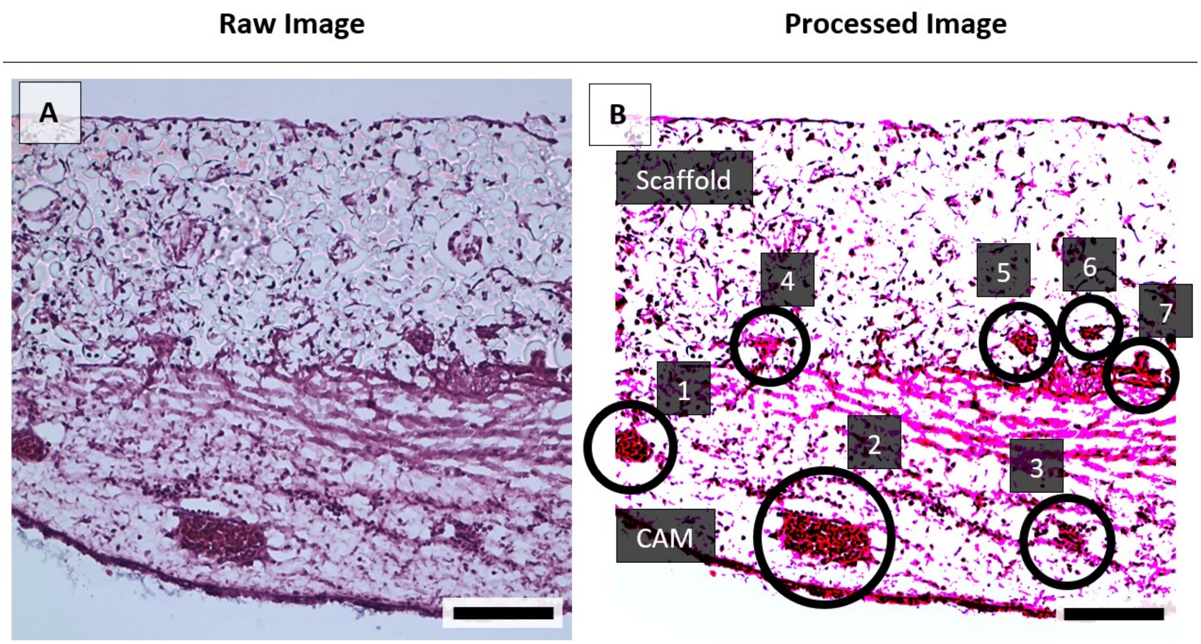

Figure S1. *An example H&E-stained image showing the colour enhancement to improve the detection of chick blood vessels in CAM: (A) inverted light microscope image acquired from histological slides (raw image) and (B) processed image via ImageJ software to increase the identifiability of the blood vessels. Red clusters of chick erythrocytes (with a black nuclei) indicate the blood vessels. Scale bars represent 100 µm.*

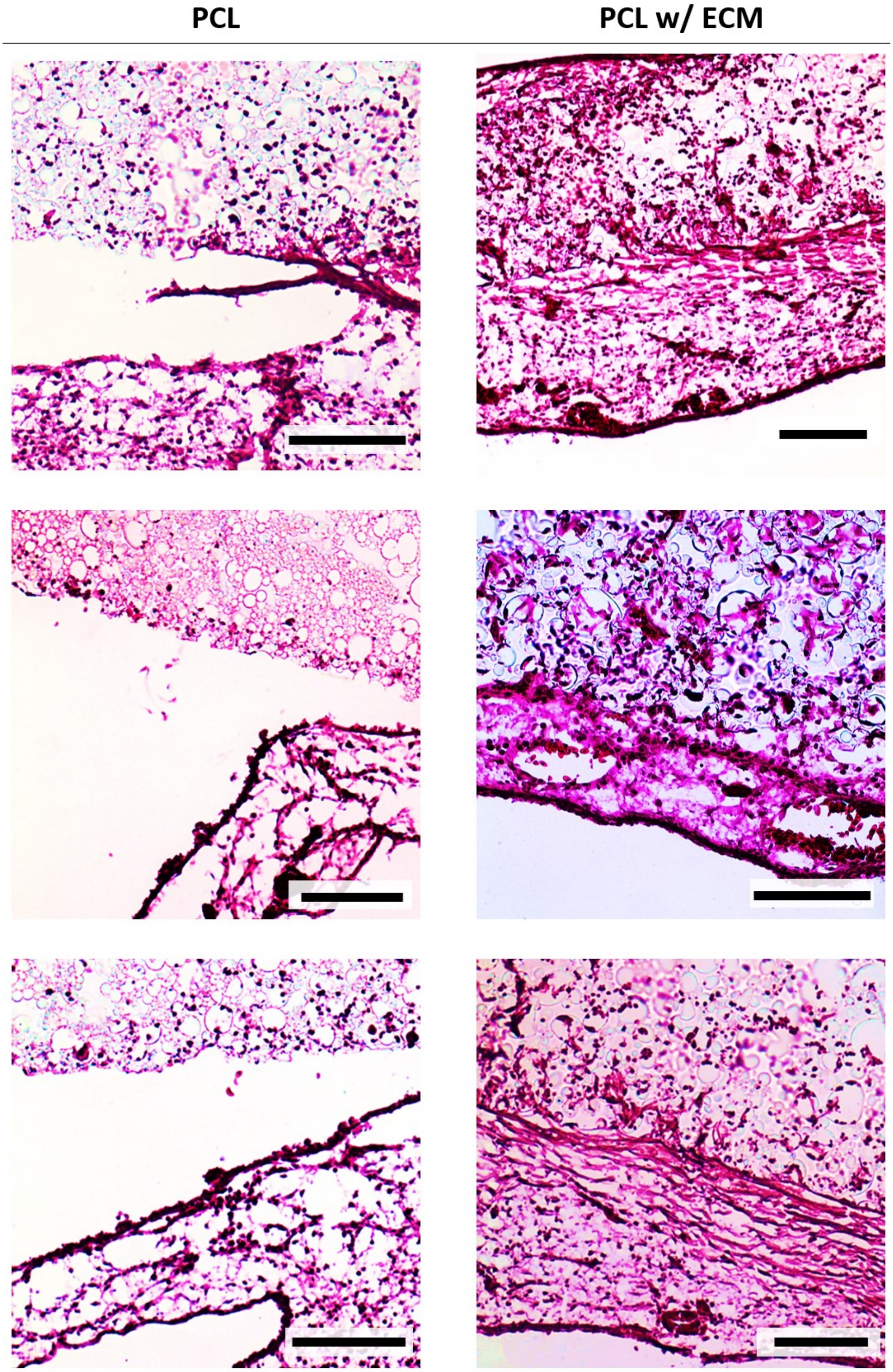

Figure S2. *Representative H&E-stained histological images that have been used for the evaluation of the angiogenic activity underneath the scaffolds of the plain PCL and bio-functionalised PCL (PCL w/ECM) scaffolds. Scale bars represent 100 μm.*

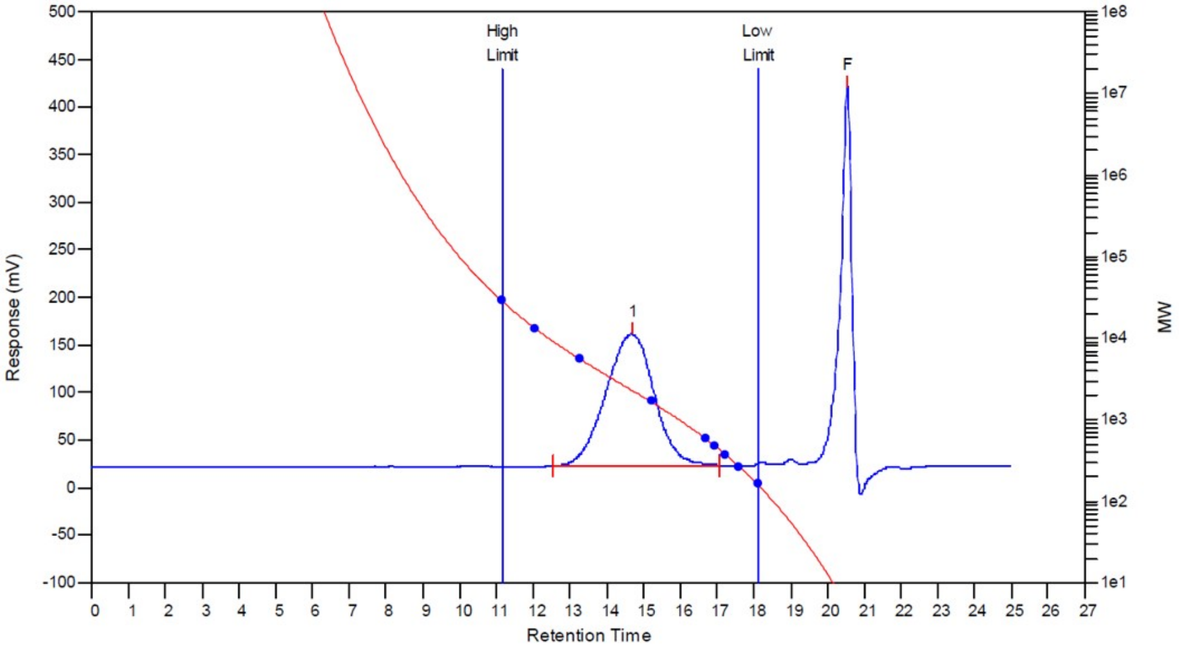

MW Averages

Peak No	*Mp*	*Mn*	*Mw*	*Mz*	*Mz+1*	*Mv*	*PD*
1	2295	2214	2556	2936	3360	2503	1.15447

Figure S3. *Gel permeation chromatography (GPC) spectrum and molecular weight (Mw) averages and polydispersity (PD) of synthesised 4PCLMA*
